# Direction-dependent differences in the quality and quantity of horizontal reaching in people after stroke

**DOI:** 10.1152/jn.00455.2022

**Published:** 2023-09-05

**Authors:** Shintaro Uehara, Akiko Yuasa, Kazuki Ushizawa, Shin Kitamura, Kotaro Yamazaki, Eri Otaka, Yohei Otaka

**Affiliations:** ^1^Faculty of Rehabilitation, Fujita Health University School of Health Sciences, Toyoake, Aichi, Japan; ^2^Department of Rehabilitation Medicine I, Fujita Health University School of Medicine, Toyoake, Aichi, Japan; ^3^Department of Rehabilitation Medicine, Takekawa Hospital, Itabashi, Tokyo, Japan; ^4^Assistive Robot Center, National Center for Geriatrics and Gerontology, Obu, Aichi, Japan

**Keywords:** cerebrovascular disorders, hemiparesis, reaching, robotics, upper extremity

## Abstract

Arm reaching is often impaired in individuals with stroke. Nonetheless, how aiming directions influence reaching performance and how such differences change with motor recovery over time remain unclear. Here, we elucidated kinematic parameters of reaching toward various directions in people with poststroke hemiparesis in the subacute phase. A total of 13 and 15 participants with mild and moderate-to-severe hemiparesis, respectively, performed horizontal reaching in eight directions with their more-affected and less-affected sides using an exoskeleton robotic device at the time of admission to and discharge from the rehabilitation ward of the hospital. The movement time, path length, and number of velocity peaks were computed for the mild group (participants able to reach toward all eight directions). In addition, the total amount of displacement (i.e., movement quantity) toward two simplified directions (mediolateral or anteroposterior) was evaluated for the moderate-to-severe group (participants who showed difficulty in completing the reaching task). Motor recovery was evaluated using the Fugl-Meyer assessment. The mild group showed worse values of movement parameters during reaching in the anteroposterior direction, irrespective of the side of the arm or motor recovery achieved. The moderate-to-severe group exhibited less movement toward the anteroposterior direction than toward the mediolateral direction at admission; however, this direction-dependent bias in movement quantity decreased, with the movement expanding toward the anteroposterior direction with motor recovery at discharge. These results suggest that direction-dependent differences in the quality and quantity of reaching performance exist in people after stroke, regardless of the presence or severity of hemiparesis. This highlights the need to consider the task work area when designing rehabilitative training.

**NEW & NOTEWORTHY** Arm reaching, a fundamental function required for the upper extremities, is often impaired after stroke due to muscle weakness and abnormal synergies. Nonetheless, how aiming directions influence performance remains unclear. Here, we report that direction-dependent differences in the quality and quantity of reaching performance exist, surprisingly regardless of the presence or severity of hemiparesis. This result highlights the need to consider the task work area when designing rehabilitative training.

## Graphical abstract

**Figure:**
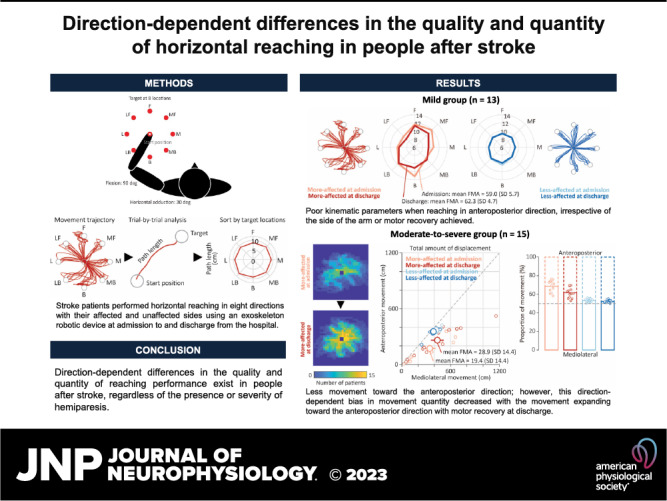

## INTRODUCTION

Approximately 80% of people after stroke suffer from upper-extremity hemiparesis (1). Hemiparesis impairs reaching, a fundamental function of the upper extremity, which considerably affects activities of daily living and quality of everyday life.

As reaching movement consists of multijoint movements, particularly in the shoulder and elbow, it requires temporally and dynamically coordinated/synergistic exertion of related muscle activities. Due to deficits underlying poststroke hemiparesis in the contralesional upper extremity such as muscle weakness (2), abnormal muscle tone (3), and abnormal coactivation patterns (4, 5), kinematic properties of poststroke reaching have been characterized as an initial direction error, a shortening of movement amplitudes, slowness in speed, and abnormal coordination of multijoint movement (6–12). It has also been suggested that people with stroke have motor deficits, including abnormal reaching kinematics, even in the ipsilesional upper extremity (13–16). Previous studies showed that some kinematic parameters of reaching were associated with the level of motor impairment in the affected upper extremity (8, 11, 12, 17, 18) and exhibited a favorable change along with improvement in hemiparesis (12, 17, 19, 20), suggesting that poststroke abnormal reaching is attributed to deficits in hemiparesis. In particular, movement metrics captured during reaching on a two-dimensional horizontal plane can better reflect motor control improvement per se while minimizing the contaminating effects of antigravity strength and compensation (21–23).

Abnormal kinematic properties of poststroke reaching and their relative changes in conjunction with motor recovery have been widely elucidated. Nonetheless, whether and how aiming directions influence the quality and quantity of reaching remain unclear. Although previous reports have shown poststroke abnormal kinematic properties in horizontal reaching (12, 17, 18, 24, 25), direction-dependent differences in kinematics have not been elucidated because many previous studies collated movements in all directions as part of their analysis and masked directional differences (17–20) or compared reaching only in the forward directions away from the body (12, 24, 25). As is well known, reaching toward different directions engages various patterns of muscle activities and coordinated multijoint movements (26). Therefore, it is conceivable that aiming directions substantially affect reaching performance in poststroke hemiparesis and that this potential direction-dependent pattern of reaching abnormalities shows a favorable change with motor recovery.

The present study aimed to explore direction-dependent differences in the movement properties of poststroke reaching and their potential changes in conjunction with improvement in motor impairment. To this end, we asked people with stroke in the subacute phase to perform a gravity-supported, planar, rapid-reaching task toward eight different directions with their more-affected and less-affected sides at admission and discharge during their hospitalization. Given that reaching performance significantly differs (i.e., able or unable) depending on motor impairment severity, we classified participants into mild and moderate-to-severe hemiparesis groups according to the level of motor impairment severity. The presence of direction dependency was assessed by movement properties representing the quality or quantity of movement separately for each group. Note that we refer to the contralesional and ipsilesional sides as the “more-affected” and “less-affected” sides in the present study because of the possibility that the ipsilesional side might not be completely intact even though motor impairment on the ipsilesional side was not specifically tested.

## MATERIALS AND METHODS

### Participants

The study protocol was approved by the Ethics Review Committee of Fujita Health University (Approval No. HM18-232) and conformed to the 1964 Declaration of Helsinki, as revised in 2013. A total of 28 people with poststroke hemiparesis in the subacute phase [mean age, 63.5 yr; standard deviation (SD), 12.4; 5 females] participated in this study. All participants were recruited by convenience sampling from a rehabilitation ward in Fujita Health University Hospital between April 2018 and February 2021 and were provided with rehabilitation training (including physical, occupational, and speech-language therapies) for 3 h a day at maximum, 7 days a week, during their hospitalization. All participants provided written informed consent before their participation in the study. We included participants if they met the following inclusion criteria: *1*) impairment in the unilateral upper extremity due to first-ever ischemic or hemorrhagic stroke, *2*) ability to maintain the sitting position, and *3*) sufficient cognition to understand a task. The exclusion criteria were as follows *1*) inability to maintain the sitting position even with the backrest and *2*) inability to follow instructions by medical professionals.

### Arm-Reaching Task

The participants’ arm-reaching performance was assessed at two different time points, namely, at admission to the rehabilitation ward (mean, 38.1 days after onset [SD, 15.9]) and at discharge from the rehabilitation ward (mean, 76.2 days [SD, 36.5]). The participants performed reaching movement on the KINARM exoskeleton robotic device (BKIN Technologies, Kingston, Canada). This robotic device mechanically provides full gravitational support for the arms, forearms, and hands with troughs and permits only horizontal motion involving shoulder horizontal adduction and abduction as well as elbow flexion and extension. A securing strap was used to hold the trunk to the chair. The participants’ reaching movement was monitored using the “Visually-Guided Reaching Task” (11), one of the standard tasks implemented in the KINARM, with a temporal and spatial resolution of 1 kHz and 0.01 cm, respectively. All participants performed the task with the less-affected arm first and subsequently with the more-affected arm during each period.

**Figure 1.**
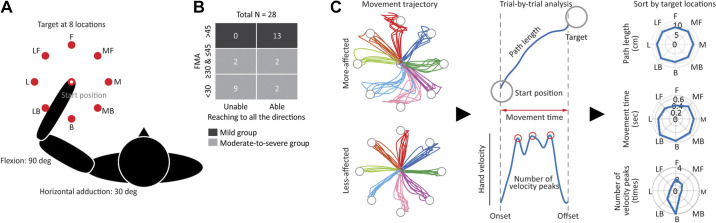
Reaching task, participant groups, and movement parameters of reaching. *A*: starting position and target locations. The participants performed arm-reaching movement on a two-dimensional horizontal plane toward one of eight targets appearing in the medial (M), medial forward (MF), forward (F), lateral forward (LF), lateral (L), lateral backward (LB), backward (B), and medial backward (MB) directions from the starting position. *B*: classification of the participants into two groups. The participants were classified into the mild and moderate-to-severe groups according to the level of motor impairment severity, as evaluated using the Fugl-Meyer assessment for upper extremity (FMA-UE), a clinical assessment score for upper-extremity impairment. The numbers inside indicate the number of participants included in each category. *C*: hand paths and three movement parameters in a representative participant in the mild group. All right-side data, regardless of whether they were data for the more-affected or less-affected side, were flipped to correspond to the left-side data.

In this task, the participants were required to quickly and accurately make a reaching movement from a centrally located visual target (a red circle with 1.0-cm radius) to one of eight peripheral targets (a red circle with 1.0-cm radius) distributed uniformly on the circumference of a circle at 10 cm from the central target by moving the shoulder and elbow (Fig. 1*A*). The hand position was tracked by a computerized representation (a white circle with 0.4-cm radius) of the index finger’s tip. The central target was set near the center of the arm’s workspace (90° of elbow flexion and 30° of shoulder horizontal adduction). The trials commenced when the white circle representing the hand position was held within the central target for 1,250–1,750 ms, leading to the appearance of a peripheral target. Upon presentation of a target, the participants started to move their hand toward the illuminated target within a limited time of 3,000 ms. Subsequently, the central target appeared and then moved on to the next trial. The participants repeated a total of 64 trials in which eight peripheral targets were randomly presented so that every set of eight consecutive trials included one of each of the target locations. If the participants failed to move the white circle back within 5,000 ms or were unable to hold it within the central target for a certain period, the counter of the number of trials increased without target appearance.

### Participant Groups

We classified the participants into two groups according to the level of motor impairment severity, as evaluated using the Fugl-Meyer assessment for upper extremity (FMA-UE), which defines >45 as mild, ≥30 and ≤45 as moderate, and <30 as severe (27): those who were defined as “mild” were assigned to the mild group (FMA-UE score range, 46–66; *n* = 13) and those who were defined as “moderate” or “severe” were assigned to the moderate-to-severe group (score range, 3–45; *n* = 15) [Table 1, Supplemental Table S1 (all Supplemental material is available at 10.5281/zenodo.8223050), and Fig. 1*B*]. This was done because reaching performance differed significantly depending on the severity of the motor impairment; some participants were able to move their hand to reach targets in all directions, whereas others were unable to reach targets. In fact, 11 out of 15 participants in the moderate-to-severe group were unable to perform reaching in all directions, whereas all in the mild group were able to do so.

**Table 1.** Participants’ demographic data

Variable	Mild Group (*n* = 13)		Moderate-to-Severe Group (*n* = 15)	
	Admission	Discharge	Admission	Discharge
Age, means (SD)	65.2 (14.8)		62.1 (10.1)	
Sex				
Male, *n* (%)	11 (84.6%)		12 (80.0%)	
Female, *n* (%)	2 (15.4%)		3 (20.0%)	
Type of stroke				
Infarction, *n* (%)	9 (69.2%)		7 (46.7%)	
Hemorrhage, *n* (%)	4 (30.8%)		8 (53.3%)	
More-affected side				
Left, *n* (%)	10 (76.9%)		8 (53.3%)	
Right, *n* (%)	3 (23.1%)		7 (46.7%)	
Time since stroke				
Mean days (SD)	34.6 (15.8)	55.6 (16.6)	41.1 (15.8)	94.1 (39.9)
FMA-UE				
Mean score (SD)	59.0 (5.7)	62.3 (4.7)	19.4 (14.4)	28.9 (14.4)

FMA-UE, Fugl-Meyer assessment for upper extremity; SD standard deviation.

### Movement Parameters of Reaching

We analyzed the movement parameters of reaching separately for the two groups.

#### Movement time, path length, and number of velocity peaks.

The mild group comprised participants who were able to perform reaching toward all eight directions with at least one trial (Fig. 1*B*). For the mild group, we characterized reaching performance using the following three movement parameters from each trial: movement time, hand path length, and number of positive peaks in the hand velocity (Fig. 1*C*). Movement time and hand path length, which are the most popular indices as total metrics, refer to the total elapsed time and length traveled from movement onset to offset, respectively (28, 29). The number of velocity peaks is an index for the smoothness of movement, counted as the number of positive peaks in the hand velocity between movement onset and offset (19, 20, 28–30). To detect the movement onset, we first computed two statistical thresholds based on the hand speed of each participant (11), namely, the lower speed threshold (i.e., the median hand speed across all trials for 500 ms before illumination of a peripheral target) and the upper-speed threshold (i.e., the 95th percentile of hand speed during this same time period). Subsequently, we identified the time, after the presentation of a peripheral target, when the hand started to move and leave the central target. Finally, we reviewed the timeframe of this hand movement in reverse to identify the first instance of one of the following: *1*) a local minimum in hand speed that was below the upper-speed threshold or *2*) a decrease in hand speed below the lower speed threshold. We defined this time point as the movement onset. Movement offset was detected as the time when the hand (i.e., the white circle) entered a peripheral target. For the number of velocity peaks, hand velocity was filtered using a sixth-order double-pass Butterworth filter with a cutoff frequency of 10 Hz, and the number of positive peaks was obtained by detecting local peaks (i.e., data samples larger than their neighboring samples) from the filtered velocity profiles without using specific thresholds such as minimum height and distance.

#### Total displacement toward the anteroposterior and mediolateral directions.

Considering that 11 out of 15 participants in the moderate-to-severe group showed difficulty in completing the reaching task for at least one of the eight peripheral targets using the more-affected side (Fig. 1*B*), the abovementioned parameters could not be adopted as proxies for the assessment of direction-dependent differences in reaching performance. In addition, some participants with severe hemiparesis exhibited only a little amount of movement outside the central target; even so, this could be observed only in a few trials. Therefore, irrespective of at which location a target appeared, we focused on how much movement they were able to exert (i.e., quantity of movement) in either the anteroposterior (AP_total_) or mediolateral direction (ML_total_). For this, we broke down point-to-point (i.e., per millisecond) movements into those toward the anteroposterior and mediolateral directions from time *t* to time *t* + 1 and then computed the total unsigned displacement for each direction (e.g., ${\text{AP}}_{total}=\sum_{t=1}^{n-1}\left|\left(AP_{t+1}-AP_{t}\right)\right|$). This was only performed for trials in which the white circle representing the hand position successfully left the central target. Data from all qualified trials were pooled within a participant.

### Interjoint Coordination Patterns

To understand the interjoint coordination patterns of the shoulder and elbow joints while reaching toward each of the eight directions, we visualized the time-course changes in each joint angle. Because the data length (i.e., movement time required for reaching) could be different among trials within a participant and between the participants, the data on joint angle in each trial were resampled to be expressed with 0–100% of movement.

### Upper-Extremity Motor Impairment

The level of motor impairment was assessed using the FMA-UE at admission and discharge.

### Data Analysis

For the mild group, we first computed the mean movement time, path length, and number of velocity peaks for each direction. Trials in which the participants failed to hold the white circle representing hand position within the central target (i.e., a peripheral target did not appear) or failed to reach a peripheral target within 3,000 ms were excluded from the analysis. The mean percentage of excluded trials for the more-affected side was 9.6% (SD, 14.2%) at admission and 7.1% (SD, 8.3%) at discharge. To analyze all participants with left and right hemiparesis together, the right-side data were flipped to correspond to the left-side data. For the analysis of differences in movement metrics depending on aiming directions between the more-affected and less-affected sides and expected motor recovery during hospitalization, we performed a three-way repeated-measures analysis of variance (ANOVA_RM_) for each parameter with within-participant factors of direction (eight directions), side (more-affected and less-affected), and time (admission and discharge).

For the moderate-to-severe group, we first investigated the presence of directional predominance in the exerted movement on the more-affected side between the anteroposterior and mediolateral directions, as well as its potential changes in conjunction with motor recovery. As in the mild-to-moderate group, we excluded trials in which the participants failed to keep the white circle representing hand position within the central target. However, we included trials in which a peripheral target appeared and the participant’s hand successfully left the central target, even if the hand failed to reach this target. The mean percentage of excluded trials for the more-affected side was 65.1% (SD, 34.3%) at admission and 41.5% (SD, 38.0%) at discharge. To control substantial differences in the amount of exerted movement among participants, each of the anteroposterior (AP_total_) and mediolateral (ML_total_) displacements were represented as a proportion of the sum of them for each participant (e.g., *AP_total_ = AP_total_/*(*AP_total_ + ML_total_*) × 100). Considering missing data for some participants who were unable to perform even one trial (admission, *n* = 3; discharge, *n* = 1, all were different individuals and no identical participant data were excluded), we implemented a linear mixed-effects model for the proportion of mediolateral displacement, with side (more-affected and less-affected) and time (admission and discharge) as the fixed effects and with participant as a random effect to assess the significance of the fixed effects.

A paired *t* test was applied to the FMA-UE scores at admission and discharge to assess the improvement in upper-extremity motor impairment during hospitalization. All statistical analyses were performed using SPSS version 26 (IBM Corp., Armonk, NY). All ANOVA_RM_ were tested for the assumption of homogeneity of variance with Mauchly’s test of sphericity. For those tests in which this assumption was violated, the Greenhouse–Geisser correction statistic was applied. Effects were considered statistically significant if *P* < 0.05.

## RESULTS

### Upper-Extremity Motor Recovery during Hospitalization

The participants’ demographic data are presented in Table 1 and Supplemental Table S1. Both groups exhibited a significant improvement in upper-extremity motor impairment, as revealed by a greater FMA-UE score at discharge than at admission (mild: *t*_12_ = 2.49, *P* = 0.028; moderate-to-severe: *t*_14_ = 5.45, *P* < 0.001).

### Poor Movement Parameters in the Anteroposterior Direction, Regardless of the Side of the Arm or Motor Recovery in the Mild Group

**Figure 2.**
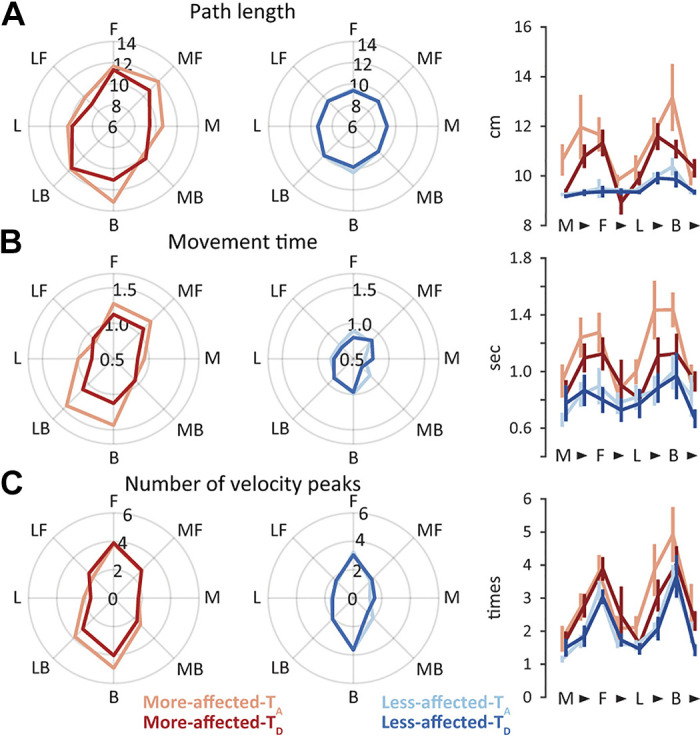
Direction-dependent differences in movement parameters in the mild group. Path length (*A*), movement time (*B*), and number of velocity peaks (*C*). Group means for the more-affected and less-affected sides at admission (More-affected-T_A_ and Less-affected-T_A_) and discharge (More-affected-T_D_ and Less-affected-T_D_) are displayed in the left and right spider plots, respectively. The rightmost panels with line graphs represent the group means and standard errors of means for each movement parameter when reaching toward the medial (M), medial forward (MF), forward (F), lateral forward (LF), lateral (L), lateral backward (LB), backward (B), and medial backward (MB) directions.

**Figure 3.**
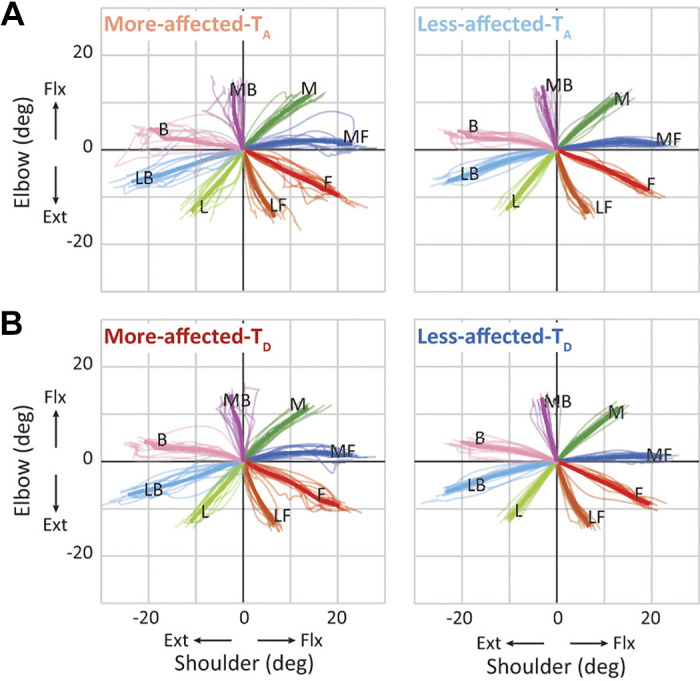
Patterns of joint movement in the mild group. The interjoint coordination patterns of the more-affected and less-affected sides at admission (More-affected-T_A_ and Less-affected-T_A_) (*A*) and at discharge (More-affected-T_D_ and Less-affected-T_D_) (*B*). The delta angle in the shoulder and elbow joints while reaching toward the medial (M), medial forward (MF), forward (F), lateral forward (LF), lateral (L), lateral backward (LB), backward (B), and medial backward (MB) directions is presented in the *x*- and *y*-axes, respectively. Thick and thin colored lines indicate the mean between participants and the mean within a participant, respectively. To control the difference in data length among trials within a participant and between the participants, the data in each trial were resampled to be expressed with 0–100% of movement. Note that the angle (0, 0) represents the starting arm position.

In the mild group, all three movement parameters showed greater values on the more-affected side than on the less-affected side (significant main effect of side, *P* < 0.01 for all three parameters; Fig. 2). Regardless of these global differences, all three parameters had greater values when reaching toward targets located in the anteroposterior direction, as found in vertically long squares in spider plots (significant main effects of direction, *P* < 0.001 for all three parameters). Specifically, the movement time and number of velocity peaks were similar in shape between the more-affected and less-affected sides (no significant direction × side interaction, *P* > 0.05; Fig. 2, *B* and *C*). When visualizing the interjoint coordination patterns between the shoulder and elbow joints during reaching toward each direction (Fig. 3), we observed that the movement parameters seemed to be poor when reaching toward directions requiring a relatively large amount of movement in the shoulder joint (forward, medial forward, backward, and lateral backward). Importantly, the direction-dependent differences in these values were consistent over time (no significant direction × time interaction, *P* > 0.1; no significant direction × side × time interaction, *P* > 0.1 for all three parameters), indicating that these patterns did not substantially change even with the recovery of the level of impairment in the more-affected upper extremity. All statistical results are summarized in Table 2. Collectively, these results suggested the presence of a direction-dependent difference in the quality of horizontal reaching among patients with mild hemiparesis, irrespective of the side of the arm or motor recovery over time.

**Table 2.** ANOVA_RM_ for movement parameters in the mild group

	Path Length			Movement Time			Velocity Peaks		
Factor	df	*F*	*P*	df	*F*	*P*	df	*F*	*P*
Direction	3.4, 41.0	8.32	<0.001	3.7, 45.3	10.91	<0.001	3.6, 43.6	22.06	<0.001
Side	1, 12	14.16	0.003	1, 12	12.27	0.004	1, 12	9.34	0.01
Time	1, 12	3.09	0.104	1, 12	0.97	0.344	1, 12	0.18	0.678
Direction × Side	3.3, 39.8	3.17	0.030	3.6, 43.4	2.24	0.086	3.0, 35.4	1.07	0.373
Direction × Time	3.0, 35.6	1.48	0.237	3.4, 40.6	0.85	0.489	4.0, 47.7	1.11	0.363
Side × Time	1, 12	2.54	0.137	1, 12	1.93	0.190	1, 12	0.49	0.496
Direction × Side × Time	10.2, 144.5	0.84	0.490	2.9, 35.3	1.72	0.182	7, 84	0.75	0.633

### Small Amount of Movement toward the Anteroposterior Direction and Its Increase with Motor Recovery in the Moderate-to-Severe Group

**Figure 4.**
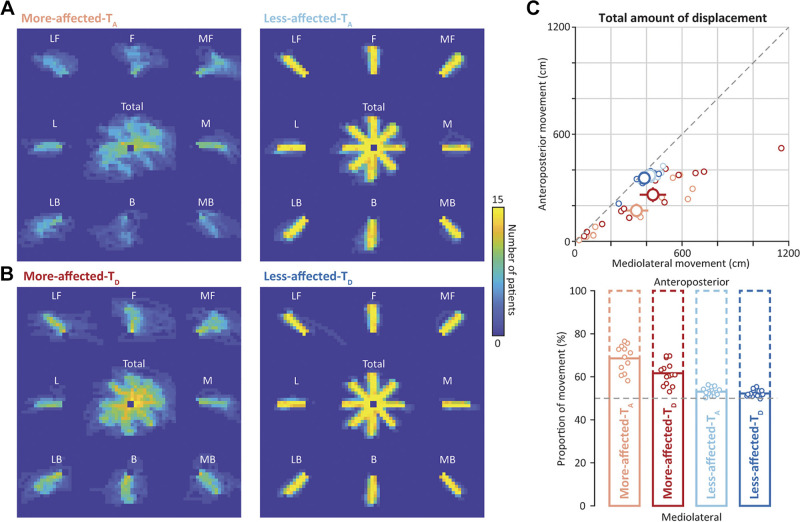
Quantity of reaching movement in the moderate-to-severe group. The hand path while reaching with the more-affected and less-affected sides at admission (More-affected-T_A_ and Less-affected-T_A_) (*A*) and at discharge (More-affected-T_D_ and Less-affected-T_D_) (*B*). The hand path is superimposed among participants and visualized in a two-dimensional histogram colormap for display purposes. The center colormap represents the hand path when all qualified trials were included regardless of the target location, whereas the surrounding ones represent the hand path when a target appeared at each location: medial (M), medial forward (MF), forward (F), lateral forward (LF), lateral (L), lateral backward (LB), backward (B), and medial backward (MB). *C*: total amount of displacement toward the mediolateral (ML_total_ in the *x*-axis) and anteroposterior (AP_total_ in the *y*-axis) directions. The *top* and *bottom* show the absolute and relative (proportion of the total) values. Small dots in the *left* represent the individual data of the more-affected side at admission (right red) and discharge (red) as well as those of the less-affected side at admission (right blue) and discharge (blue). Large dots with error bars represent the means and standard errors of means across participants. Bar graphs in the *bottom* represent the mean proportion of the amount of movement toward the mediolateral direction. Small dots represent individual data.

As found in the superimposed hand path, reaching movement using the more-affected side in the moderate-to-severe group tended to appear more in the mediolateral direction than in the anteroposterior direction at admission (Fig. 4*A*). Subsequently, the movement appeared to expand toward the anteroposterior direction along with motor recovery at discharge (Fig. 4*B*). These qualitative direction-dependent differences in reaching performance were not confirmed on the less-affected side in which the hand path converged into straight lines (Fig. 4, *A* and *B*).

Quantitative evaluation of reaching revealed that the total amount of movement on the more-affected side was substantially increased toward both the anteroposterior (mean, 261.1 cm [SD, 153.7]) and mediolateral (mean, 436.0 cm [SD, 295.9]) directions at discharge, compared with admission (mean of 172.2 cm [SD, 135.2] for the anteroposterior direction and 343.4 cm [SD, 246.9] for the mediolateral direction), indicating global increases in movement capacity (Fig. 4*C*, top). Importantly, with respect to the proportion of movements toward each of the mediolateral and anteroposterior directions, the more-affected side showed decreased movements toward the anteroposterior direction (mean of 68.6% [SD, 6.3] for the mediolateral direction and 31.4% [SD, 6.3] for the anteroposterior direction); nevertheless, such movements toward the anteroposterior direction increased at discharge (mean of 61.7% [SD, 5.4] and 38.3% [SD, 5.4]). In contrast, those on the less-affected side remained constant over time (mean of 53.1% [SD, 1.8] and 46.9% [SD, 1.8] at admission vs. mean of 52.3% [SD, 1.5] and 47.7% [SD, 1.5] at discharge; significant side × time interaction, *P* < 0.01; Table 3 and Fig. 4*C*, *bottom*). Note that the number of included trials was comparable across directions, even though a relatively large number of trials on the more-affected side were excluded for this analysis (Supplemental Fig. S1). These results indicated that, in the moderate-to-severe group, a propensity in the movement of the more-affected side toward the mediolateral direction existed in the earlier stage after stroke; however, the degree of this propensity changed along with improvement in motor impairment such that the proportion of movement expanded toward the anteroposterior direction. All statistical results are summarized in Table 3.

**Table 3.** Linear mixed-effects model for movement parameters in the moderate-to-severe group

	Proportion of mediolateral movement		
Factor	df	*F*	*P*
Side	1, 33.6	161.33	<0.001
Time	1, 34.0	13.48	0.001
Side × Time	1, 34.0	8.57	0.006

## DISCUSSION

The present study explored direction-dependent differences in the quality and quantity of reaching movement after stroke and their potential changes in conjunction with motor recovery. In participants with mild hemiparesis who were able to perform the center-out reaching task, a comparable pattern of direction-dependent difference in movement quality was confirmed on both the more-affected and less-affected sides; while movement toward the anteroposterior direction tended to be worse than that in the mediolateral direction, the performance on the more-affected side was generally worse than that on the less-affected side. This pattern of direction-dependent difference did not substantially change, even with upper-extremity motor recovery. In participants with moderate-to-severe hemiparesis who experienced difficulty in sufficiently performing the reaching task, a direction-dependent bias (mediolateral > anteroposterior) in movement quantity was initially observed on the more-affected side at admission. Nevertheless, this direction propensity decreased with motor recovery at discharge.

We found that the quality of horizontal reaching on the more-affected side tended to be worse than that of the less-affected side. This finding is consistent with the results of several studies reporting abnormal kinematics in poststroke reaching (6–12). With a task involving horizontal reaching toward various directions uniformly distributed on the circumference, the present study further showed that reaching performance changed depending on aiming direction, that is, poor performance was observed in reaching attempts toward and away from the participants’ own body (i.e., the anteroposterior direction). This result elaborated a previous finding in poststroke hemiparesis that revealed direction-dependent differences in the performance of horizontal reaching movements away from the body (12, 24).

However, we surprisingly found a comparable pattern of direction-dependent difference in performance on the less-affected side as well; additionally, we observed that the pattern did not substantially change with improvement in motor impairment. Given that poststroke hemiparesis tends to exhibit abnormal muscle activities that lead to movement disruption at the level of interjoint coordination (4, 6, 31), it was highly plausible to expect the presence of direction-dependent difference in performance specific to the more-affected side. Moreover, it was expected that this pattern of direction dependency would change with recovery from motor impairment, accompanied by a decrease in synergistic muscle activity patterns evaluated by the FMA (32, 33). One possible explanation for these unexpected results may be that the participants in this study conducted the horizontal reaching task with the arm fully supported by troughs and/or their arm position was constrained at 90° of shoulder abduction/flexion, which may not allow the present results to be directly generalized. Previous studies have shown that abnormal muscle activity patterns tend to emerge more while attempting to move the more-affected arm under the influence of gravity (34, 35). In the present study, having the arm fully supported during the reaching task may have minimized the influence of abnormal muscle activity patterns, allowing us to evaluate a purer aspect of motor control in poststroke hemiparesis (21–23). These factors may have contributed to the present finding that no paresis-specific pattern of direction-dependent difference in reaching performance existed in people with mild hemiparesis. Another possibility is that direction-dependent difference found in the present study is specific to people with stroke. Given that some people with subacute stroke may also have abnormal motor function in the ipsilesional upper extremity (13–16), the comparable pattern of direction-dependent difference in reaching performance on both sides may be associated with a potential motor deficit present on both sides, even if its magnitude is different.

The comparable pattern of direction-dependent difference in performance on both the more-affected and less-affected sides may reflect the presence of innate biomechanical features in arm reaching. A previous report revealed the existence of direction-dependent difference in the kinematics of horizontal reaching: reaching velocity tended to be slower while reaching in the direction along the forearm axis than in the direction orthogonal to the forearm axis (7). As this was commonly found in healthy and poststroke participants, irrespective of dominant/nondominant or more-affected/less-affected sides (7), this kinematic difference was thought to be attributed to the inertial anisotropy of the limb, which affects reaching speed (36, 37). In addition, it was shown in healthy volunteers that the ease of movement might differ, depending on aiming directions, potentially resulting in a different amount of biomechanical cost (38, 39). When horizontal reaching is freely performed toward any direction, preferred reaching tends to converge into four directions: two longitudinal directions along the forearm axis involving active shoulder and passive elbow motions and two transverse directions orthogonal to the forearm axis involving active elbow and passive shoulder motions (38, 39). These propensities were likely associated with a tendency to perform a simplified pattern of shoulder and elbow movement, during which one of the two joints was actively rotated, whereas the other joint was predominantly passively rotated by interaction torque (38–40). The better reaching performance observed in the direction orthogonal to the forearm axis in the present study may be partly explained by these preferred directions with a lower biomechanical cost. However, the present study also showed that the quality of reaching in the direction along the forearm axis, which requires a relatively large amount of movement in the shoulder joint, tended to be poor. This contradiction seems to suggest that movements with lower biomechanical costs are not necessarily easy to control. In other words, there may be difficulty in controlling the smoothness of endpoint (i.e., hand) movement while reaching, which can be mainly achieved by active shoulder and passive elbow motions (such as that toward the forward, medial forward, backward, and lateral backward directions). This may be attributed to the fact that because the distance from the point of action of the main effector (shoulder muscle) to the endpoint is long, precise regulation of muscle activities would be required to control the endpoint location, even though this situation is beneficial to augmenting movement velocity.

The difference in the ease of movement depending on aiming directions may affect the quantity of exerted movement in people with moderate-to-severe hemiparesis. Supporting this perspective, those with moderate-to-severe hemiparesis tended to exert a relatively greater amount of movement toward the mediolateral direction than toward the anteroposterior direction early on after stroke. This indicates the presence of a directional bias in movement quantity and, thus, a direction-dependent difference in reaching performance among people with severe hemiparesis. Interestingly, with motor recovery, their reaching work area significantly expanded toward the anteroposterior direction in addition to the global increase in the quantity of exerted movement toward both the anteroposterior and mediolateral directions. These findings suggest that improvement in reaching performance can be represented as an increase in movement capacity toward the anteroposterior direction, even in people with severe paresis. Although the reaching work area can be expanded by an increase in compensatory trunk movement (41) and/or a decrease in the amount of effort required for lifting the limb against gravity (42), these are less likely because the present study restricted trunk movement using a fastening belt and the arm was fully supported by the troughs of the robot. It should be noted that, even if some within-participant trials were excluded in the process of analysis for the moderate-to-severe group, this would have only a marginal impact on the results showing the behavioral trend between participants as data from most participants were included in the analysis.

The present study has some limitations. First, it remains unclear how the direction-dependent difference in reaching performance changes during the chronic stage (>6 mo after onset). Second, careful caution should be exercised when interpreting the comparable pattern of direction-dependent difference between the more-affected and less-affected sides in the mild group, given that the less-affected side might also have a marginal deficit in dexterity control of reaching movement (13–16, 43). In addition, the proportion of participants with a right or left lesion was unbalanced in this group, with a greater number of participants with a right lesion. A previous study showed that individuals with a right hemisphere lesion after a stroke have similar characteristics of reach control on both the more-affected and less-affected sides (44). A future study recruiting age-matched healthy controls and people with stroke with a balanced proportion of left and right lesions should investigate whether the direction-dependent difference in reaching performance is specifically found in people with stroke and/or whether the comparable pattern of direction dependence between the more-affected and less-affected sides is specific to those with right lesions. Third, careful consideration should be given when generalizing the present results to other situations, especially when using similar reaching tasks but with different initial arm postures, because the joint coordination patterns of reaching would differ slightly depending on the initial arm position. Similarly, caution should be exercised in generalizing the present results, which were obtained in a specific experimental setting where reaching was performed with the effects of gravity eliminated. Fourth, in the present study, the data were flipped from right to left to integrate all participants with left and right hemiparesis together and to facilitate comparison between the more-affected and less-affected sides without taking into account the effects of handedness. The question of whether and how the handedness would affect the present results could be addressed with sufficient recruitment of participants. Finally, especially in the moderate-to-severe group, only a limited number of trials on the more-affected side were included in the analysis due to the severity of the motor impairment. One might think that an unbalanced number of trials included in the analysis across directions would affect the changes in the proportion of movement found in this group. However, we confirmed that the number of trials included was comparable across directions, indicating that a potential imbalance in the number of trials included would not affect the present results. Nevertheless, the present study leaves an interesting question regarding whether the expansion of the reaching work area (i.e., increased quantity of exerted movement) in people with severe hemiparesis may be related to better improvement in the quality of movement as well. To address this question, a future study needs to develop a proxy for representing quality for all levels of reaching movement, including those insufficient to complete target-directing reaching tasks.

### Conclusions

The present study showed the existence of direction-dependent differences in the quality and/or quantity of reaching performance after stroke, irrespective of the presence of hemiparesis and its level of severity. This finding highlights the need to consider the task work area when designing rehabilitative training after stroke, especially for people with severe upper-extremity hemiparesis.

## DATA AVAILABILITY

Data will be made available upon reasonable request.

## SUPPLEMENTAL DATA

10.5281/zenodo.8223050 Supplemental Table S1 and Supplemental Fig. S1: 10.5281/zenodo.8223050.

## GRANTS

This work was supported by Grants-in-Aid for Scientific Research from the Japan Society for the Promotion of Sciences [grant numbers 20K19393 (to S.U.) and 18H03135 (to Y.O.)].

## DISCLOSURES

No conflicts of interest, financial or otherwise, are declared by the authors.

## AUTHOR CONTRIBUTIONS

S.U., K.Y., E.O., and Y.O. conceived and designed research; S.U., A.Y., K.U., and S.K. performed experiments; S.U. analyzed data; S.U. and Y.O. interpreted results of experiments; S.U. prepared figures; S.U. drafted manuscript; S.U. and Y.O. edited and revised manuscript; S.U., A.Y., K.U., S.K., K.Y., E.O., and Y.O. approved final version of manuscript.
